# Supplementary figures and images for: Advancing data to care strategies for persons with HIV using an innovative reconciliation process
Source: PLoS One. 2022 May 5;17(5):e0267903. doi: 10.1371/journal.pone.0267903 (PMC9071117; doi:10.1371/journal.pone.0267903)

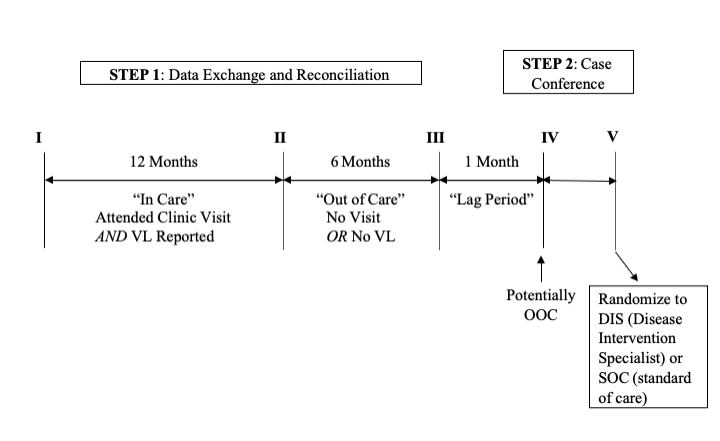

Supplement: S1 Fig — STEP 1: Data Exchange and Reconciliation (I→IV). In Care and Out of Care Definitions: “In care” patients were required to meet both of the following criteria: at least one clinic visit and an HIV VL within a designated 12-month period (I→II). “Out-of-care” patients had either no clinic visit or no VL during the subsequent six months (II→III). Data Exchange Process: Clinics and DPH transferred client-level data via a secure file transfer mechanism meeting the federal government’s security compliance requirements. Each clinic was responsible for sending data files to the DPH. The designated out-of-care period would end one month prior to when the request for data was sent, to accommodate a ‘lag period’ (III→IV) due to potential delays in HIV lab reporting. Clinics submitted two lists: (1) patients who attended a clinic visit within the 12-month date range (2) patients with no clinic visit in the subsequent 6-months. Clinic data sources included CAREWare (an electronic health system developed by the Health Resources and Service Administration (HRSA) for Ryan White Grant recipients) and Electronic Medical Record (EMR). The DPH matched data from the clinics with HIV VL data from eHARS (enhanced HIV/AIDS Reporting System), the HIV Surveillance system. Patients were further classified based on clinic visit attendance and VL data (Fig 1). Patients with a clinic visit and VL in the 6-month timeframe were excluded (Box A); the remainder were designated as “Potentially OOC” and subdivided as: Box B (no clinic visit, had VL); Box C (had clinic visit, no VL); Box D (no clinic visit, no VL). STEP 2: Case Conference (IV→V). The matched list of potentially OOC was sent back to the clinics to designate a disposition based on review of EMRs and discussion with other clinic staff. Dispositions included the following in stepwise order at time of case conference: (1) Well Patient (2 consecutive undetectable VL of ≤20 copies/ml at least 6 months apart and no evidence of detectable VL dur [file pone.0267903.s001.tiff]
